# Gut microbial dynamics and antibiotic resistance during rifaximin therapy in severe cirrhosis: a ProPILA-Rifax ancillary study

**DOI:** 10.3389/fcimb.2026.1930709

**Published:** 2026-09-29

**Authors:** Ruben Ladeira, Adrien Biguenet, Alexandre Meunier, Marion Gonthier, Vincent Di Martino, Anaïs Pitta, Edouard Bardou-Jacquet, Anne Jolivet-Gougeon, Maxime Desmarets, Thierry Thévenot, Didier Hocquet, Benoit Valot

**Affiliations:** 1 Bioinformatique et Big Data Au Service de La Santé, Oncobiologie Génétique Bioinformatique, Centre Hospitalier Universitaire (CHU) Besançon Franche-Comté, Besançon, France; 2 Université Marie et Louis Pasteur, Centre National de la Recherche Scientifique (CNRS), Unité Mixte de Recherche (UMR) 6249 Chrono-environnement, Besançon, France; 3 Hygiène Hospitalière, Centre Hospitalier Universitaire (CHU) Besançon Franche-Comté, Besançon, France; 4 Université Marie et Louis Pasteur, Institut National de la Santé et de la Recherche Médicale (INSERM), Unité Mixte de Recherche (UMR) 1098 Regulation of Immunity for therapeutic innovation in Graft, Host, Tumoral and inflammatory-associated diseases (RIGHT), Besançon, France; 5 Service d’Hépatologie, Centre Hospitalier Universitaire (CHU) Besançon Franche-Comté, Besançon, France; 6 Service d’Hépato-Gastroentérologie, Hôpital Cabrol, Reims Cedex, France; 7 Univ. Rennes, Centre Hospitalier Universitaire (CHU) Rennes, Service des Maladies du Foie, Institut National de la Santé et de la Recherche Médicale (INSERM) 1241 Nutrition, Métabolismes et Cancer (NUMECAN), Rennes, France; 8 Department of Bacteriology, Rennes University Hospital, Rennes, France; 9 Univ. Rennes, Institut National de la Santé et de la Recherche Médicale (INSERM), INRAE, Unité Mixte de Recherche (UMR) S1230 Bacterial Regulatory RNAs & Medicine (BRM), Institut Nutrition, Métabolismes et Cancer (NUMECAN) Unité Mixte de Recherche (UMR) 1241, Rennes, France; 10 Centre d’Investigation Clinique 1431, unité de METhodologie (uMETh), Institut National de la Santé et de la Recherche Médicale (INSERM), Centre Hospitalier Universitaire (CHU) Besançon Franche-Comté, Besançon, France; 11 Université Marie et Louis Pasteur, EFS, Institut National de la Santé et de la Recherche Médicale (INSERM), Unité Mixte de Recherche (UMR) 1098 Regulation of Immunity for therapeutic innovation in Graft, Host, Tumoral and inflammatory-associated diseases (RIGHT), Besançon, France

**Keywords:** antibiotic resistance, gut microbiome, gut-liver axis, liver cirrhosis, rifaximin, spontaneous bacterial peritonitis

## Abstract

**Introduction:**

Increasing evidence implicates gut dysbiosis in the pathophysiology of advanced cirrhosis. This is an ancillary study of the ProPILA-Rifax trial (NCT03069131), which evaluated rifaximin (RFX) as primary prophylaxis of spontaneous bacterial peritonitis (SBP) in patients with severe cirrhosis. Although the combined use of RFX and lactulose is effective in preventing recurrent hepatic encephalopathy (HE), its longitudinal impact on microbial communities and antibiotic resistance remains poorly characterized.

**Methods:**

We characterized longitudinal changes in the gut microbiome of 42 patients (21 RFX, 21 placebo) over 12 months using 16S rRNA gene sequencing and culture-based phenotypic susceptibility testing. Exploratory analyses between patient bacterial profiles and clinical outcomes (disease severity, overall survival, and incidence of SBP) were conducted.

**Results:**

RFX treatment reduced microbial richness and decreased the abundance of taxa typically enriched in advanced cirrhosis (e.g., *Escherichia*, *Streptococcus*, *Veillonella*). RFX increased the proportion of Escherichia coli isolates exhibiting phenotypic resistance to rifampicin, without observed cross-resistance to ciprofloxacin or cefotaxime. In exploratory analyses, a decrease in Child-Pugh scores was correlated with enrichment of commensal taxa. Moreover, higher abundances of Ruminococcus bromii and Blautia obeum at inclusion were respectively associated with improved 12-month survival and a lower incidence of SBP.

**Discussion:**

In severe cirrhosis, RFX profoundly alters gut microbial composition by reducing pathobiont abundance while promoting rifampicin resistance in *E. coli*. Furthermore, microbiota-derived signatures were associated with Child-Pugh scores, highlighting their potential to improve patient stratification.

## 1 Introduction

Liver cirrhosis is a leading cause of mortality and morbidity worldwide. The gut microbiome of cirrhotic patients is often altered by environmental and health factors, including high-fat diets and alcohol consumption. This dysbiosis can lead to intestinal barrier dysfunction, increased gut permeability and progression of liver cirrhosis (Konyn et al., 2023; Global burden of liver disease: 2023 update - Journal of Hepatology). The gut-liver axis has therefore emerged as the primary hypothesis to explain microbiota-associated complications of cirrhosis, such as hepatic encephalopathy (HE), ascites, and spontaneous bacterial peritonitis (SBP) (Hsu and Schnabl, 2023; Feng et al., 2024). However, strategies to identify microbiome-based biomarkers and therapeutic targets for modulating the gut microbiota and disease course remain limited (Albillos et al., 2020). Rifaximin (RFX), a weakly absorbed rifamycin-based antibiotic, is currently licensed for use as an add‐on therapy to lactulose for the prevention of recurrent hepatic encephalopathy (Lv et al., 2020; Yu et al., 2021; Hu et al., 2025). Recent pivotal studies have expanded our understanding of its ecological impact in advanced liver disease. For instance, the RIFSYS trial demonstrated that RFX suppresses the progressive oralization of the gut microbiome, specifically reducing the abundance of mucin-degrading pathobionts such as *Streptococcus* spp. and *Veillonella* spp (Patel et al., 2022). Moreover, the RIFA-AH study suggested clinical benefits of RFX in severe alcohol-associated hepatitis, showing a reduced incidence of infections and liver-related complications (Jiménez et al., 2022). Despite these critical insights, longitudinal analyses evaluating both microbial community changes and the emergence of antibiotic-resistant bacteria over extended prophylactic periods remain sparse. This is of particular concern given recent paradigm-shifting findings demonstrating that RFX prophylaxis can drive cross-resistance to last-resort antibiotics (Turner et al., 2024). This discovery challenges the historical assumption that RFX is a “low-risk” antibiotic for resistance development and underscores the need to monitor resistance emergence in other major gut colonizers (Shamsaddini et al., 2021). However, recent real-world data from the large global CLEARED consortium provides reassuring clinical context, demonstrating that pre-admission RFX use does not increase the risk of developing drug-resistant organism infections in hospitalized cirrhotic patients (Bajaj et al., 2026). To address these gaps, we conducted an ancillary study nested within the recently published ProPILA-Rifax trial (NCT03069131) evaluating RFX as primary prophylaxis in a highly selected cohort of patients with severe cirrhosis and low ascitic fluid protein. While the main trial did not meet its primary endpoint of improving 12-month survival, characterizing the microbial consequences of prolonged RFX exposure in this population remains of substantial scientific interest. Herein, we combined 16S rRNA sequencing and culture-based methods over a 12-month period to investigate the longitudinal impact of RFX on the gut microbiota and to assess the potential emergence of phenotypically antibiotic-resistant *Escherichia coli*, the predominant causative agent of SBP. In secondary exploratory analyses, we further investigated microbiota-derived signatures associated with liver disease severity, SBP incidence, and overall survival, irrespective of treatment allocation.

## 2 Patients and methods

### 2.1 Clinical design

The current study is the ancillary analysis of gut microbiota data from the ProPILA-Rifax study (NCT03069131), a double-blind, placebo-controlled, randomized clinical trial conducted across 17 French centers (Thévenot et al., 2025). A total of 152 subjects with severe cirrhosis, associated with grade 2 or 3 ascites and a protein level in ascites lower than 15 g/L, were included to evaluate whether RFX had a beneficial effect on 12-month survival. Subjects received either placebo (PBO) or RFX (550 mg b.i.d.) for 12 months following randomization. The trial was conducted in accordance with the Declaration of Helsinki and with the approvals of the ethics committee “CPP Sud-Méditerranée IV” under number #170709PP, and the competent authorities (French National Agency for Medicines and Health Products Safety, #170392A-42). The study also adhered to the CONSORT reporting guidelines (Moher et al., 2012).

### 2.2 Sample collection and processing

Fecal samples (n = 109) were collected from 43 randomized subjects at three centers (Besançon, Reims, and Rennes) during the course of the clinical trial. Participants received either RFX (n = 21) or PBO (n = 22) for 12 months. Samples were collected at day 1 (D1), 3 months (M3), 6 months (M6), 12 months (M12), and 18 months (M18) after patient inclusion. Samples were processed according to International Human Microbiome Standards (IHMS) consortium guidelines and stored at –80 °C until sequencing. At each time point, patients provided stool samples of approximately clementine size. Samples were stored for <4 hours under aerobic conditions, or <24 hours under anaerobic conditions, following IHMS consortium recommendations (https://human-microbiome.org/). Upon laboratory reception, samples were homogenized using a grinder (AES laboratoire, Combourg, France). A portion was divided into aliquots and stored at –80 °C for sequencing. In parallel, 10 mL of sterile physiological saline solution were added to the remaining sample, which was further homogenized. A 500 µL aliquot of the homogenate was transferred into cryotubes containing 500 µL of brain–heart infusion broth supplemented with glycerol, and stored at –80 °C for bacterial culture.

### 2.3 DNA extraction and sequencing

Metagenomic DNA was extracted from fecal samples using mechanical lysis followed by purification with the DNeasy Blood & Tissue Kit (Qiagen). Library preparation targeted the V3–V4 hypervariable regions of the 16S rRNA gene using a two-step PCR approach, and amplicons were sequenced on an Illumina MiSeq platform. DNA was extracted from 200–300 mg of fecal material, added into bead-beating tubes containing 0.1 mm glass beads and 1 mL of Inhibitex buffer (Qiagen). Mechanical lysis was performed using a FastPrep-24 instrument (MP Biomedicals) for four cycles of 45s at speed 5. DNA extraction was carried out using the DNeasy Blood & Tissue Kit (Qiagen) according to the manufacturer’s protocol. DNA concentrations were quantified using the PicoGreen dsDNA assay (ThermoFisher). Library preparation for 16S rRNA gene sequencing followed a two-step PCR approach targeting the V3–V4 hypervariable regions, using locus-specific primers 341F and 805R. The first PCR consisted of 20 cycles for amplifying, followed by a second PCR of 10 cycles for indexing (Nextera). Amplicon libraries were pooled equimolarly, and sequenced on an Illumina MiSeq platform.

### 2.4 Microbiota pre-processing and taxonomic profiling

Cleaning, filtering, and taxonomic assignment of 16S rRNA reads were performed with the nf-core/ampliseq pipeline (Straub et al., 2020), version 2.6.1. Data were trimmed based on primer sequences, and only samples with more than 2,000 reads were retained. This filtering step resulted in the exclusion of one patient from the placebo group, yielding a final cohort of 42 subjects (21 RFX, 21 PBO). Amplicon sequence variants (ASVs) were assessed by DADA2 v1.22.0 (Callahan et al., 2016) with default parameters and their taxonomic assignment was performed on the SILVA (Quast et al., 2013) database release v138. Singleton ASVs were excluded from the analysis.

### 2.5 Culture-based phenotypic quantification of antibiotic-resistant *Escherichia coli*

Total and rifampicin (RMP)-resistant *E. coli* isolates were quantified from fecal samples by culture on selective agar plates. Isolates were identified using MALDI-TOF mass spectrometry (Microflex LT; Bruker Daltonik GmbH, Bremen, Germany). We tested the stability of the resistance to RMP, and the co-resistance to ciprofloxacin and cefotaxime. To evaluate the risk of resistance emergence, we quantified the proportion of E. coli isolates resistant to rifampicin (RMP), an antibiotic structurally related to rifaximin. For each sample, total and RMP-resistant E. coli were quantified. Total E. coli concentration was determined by plating 100 µL of serially diluted samples on Tryptone Bile X-Glucuronide (TBX) agar, followed by overnight incubation at 44 °C. Blue colonies, indicative of E. coli, were then counted. RMP-resistant E. coli were quantified by plating the same dilutions on Muller-Hinton agar (MHA) containing 100 mg/L RMP, incubated overnight at 37 °C. All morphotypes of colonies were identified using MALDI-TOF mass spectrometry (Microflex LT; Bruker Daltonik GmbH, Bremen, Germany) according to the manufacturer’s recommendations. To test the stability of their resistance to RMP and to assess whether RMP-resistant E. coli were co-resistant to clinically important antibiotics, isolates (between 1 and 5 per fecal sample) were subcultured on MHA overnight at 37 °C, then streaked onto MHA supplemented with 100 mg/L of RMP, 2 mg/L of ciprofloxacin, or 8 mg/L of cefotaxime. Each sample was analyzed as a single replicate.

### 2.6 Statistical analysis

#### 2.6.1 Assessment of RFX effect on microbiota alpha richness and diversity

Count tables were normalized using Scaling with Ranked Subsampling (SRS) (Beule and Karlovsky, 2020). Diversity metrics (microbial richness and Shannon index) were computed using ASVs as species proxies with the phyloseq (v1.46.0) R package. Longitudinal changes in these metrics were assessed using an aligned rank transform (ART) mixed-effects model, testing the effect of time within each treatment arm while accounting for subject-level random effects. *Post-hoc* contrasts between inclusion and follow-up visits were performed on the aligned ranks using the art.con function. To formally compare longitudinal trajectories between treatment arms, complementary ART mixed-effects models including treatment arm, time, their interaction, and subject as a random effect were fitted for Fisher richness and Shannon diversity during the treatment period (D1–M12). Planned treatment-by-time interaction contrasts compared changes from D1 to M3, M6, and M12 between the RFX and PBO arms. All *post-hoc P*-values were adjusted for multiple testing using the FDR procedure.

#### 2.6.2 Assessment of RFX effect on microbiota communities

Changes in microbial community composition between inclusion (D1) and follow-up visits (M3, M6, M12) were assessed within each treatment arm, excluding M18 due to treatment discontinuation. Group-level differences in community composition estimated by Weighted UniFrac distances were tested using permutational multivariate analysis of variance (permANOVA). To account for the longitudinal study design, permutations were restricted within subjects. To assess the effect of RFX on species-level abundances, zero-inflated generalized linear mixed models were fitted with a negative binomial distribution (Yang and Chen, 2023; McGillycuddy et al., 2025), including subject as a random effect and sequencing depth as an offset. Global significance for each ASV was assessed using Type II Wald Chi-square tests. As a complementary analysis, raw counts were aggregated at the genus level and analyzed using zero-inflated negative-binomial generalized linear mixed models including treatment arm, time, their interaction, sequencing depth as an offset, and subject as a random effect. Planned treatment-by-time interaction coefficients compared D1-to-follow-up changes between treatment arms at M3, M6, M12, and M18 and were assessed using Wald z-tests. All *post-hoc P*-values were adjusted for multiple testing using the FDR procedure.

#### 2.6.3 Assessment of the effect of RFX treatment on *E. coli* RMP-resistance

Fisher’s exact test was performed to assess the effect of RFX on the presence/absence of RMP-resistant *E. coli* mutants for each treatment arm. Among RMP-resistant *E. coli*-positive samples, an ART model estimated the difference in the proportion of RMP-resistant *E. coli* between inclusion and follow-up for each treatment arm, taking into account the random effect of subjects.

#### 2.6.4 Exploratory analyses of bacterial biomarkers associated with clinical outcomes

Exploratory analyses linking microbial taxa abundances to clinical outcomes were conducted as subsequent hypothesis-generating steps. First, an evaluation of association between microbial composition and Child-Pugh score was performed by permANOVA using the Child-Pugh score as the explanatory variable, with permutations constrained by subject. Then, species-level associations with disease severity were assessed using species-wise ordinal generalized linear mixed models, fitted independently for each taxon. Microbial abundances were transformed using the centered log-ratio (CLR) to account for the compositional nature of microbiome data. In each model, the Child-Pugh score was specified as the response variable, CLR-transformed microbial abundance as a fixed effect, treatment arm as a covariate, and subject as a random effect. Time was not incorporated into the model because patient dropout resulted in collinearity between Child-Pugh scores and time; therefore, Child-Pugh was modeled as the sole dependent variable with CLR-transformed microbial abundance as the primary explanatory variable. *P*-values were adjusted for multiple testing using the FDR. Each species significantly associated with Child-Pugh scores was subsequently tested for its association with clinical outcomes. Subjects were first stratified into prognostic groups based on microbial abundance at inclusion using a grid-search procedure over candidate cutoffs derived from the empirical distribution and constrained to include at least 30% of subjects in each group (Derosa et al., 2022). The cutoff yielding the lowest Akaike Information Criterion (AIC) was selected (Sutherland et al., 2023). For SBP, competing risk analyses were performed using Fine–Gray models, with death considered as a competing event. Overall survival was analyzed using Cox proportional hazards models. Treatment arm was included as a stratification variable to evaluate its effect on these associations. All tests were two-sided, and statistical significance was defined as *P* < 0.05. Statistical analyses were performed using R software (v4.3.1).

## 3 Results

### 3.1 Baseline characteristics of the study cohort

Among the 152 subjects included in the modified intention-to-treat population (Thévenot et al., 2025), 43 subjects from three centers provided fecal samples and were included in the present study. A total of 107 samples from 42 subjects meeting sequencing quality control criteria were retained for subsequent analyses (**Figure 1**). At inclusion, the median (IQR) age was 59.3 (Q1-Q3: 52-70) years, the median Child-Pugh score was 11.0 (10.0-12.0), and 80.9% of the subjects were male. During follow-up, ten subjects died, and two were lost to follow-up. In this sub-cohort, nine patients developed SBP. Before initiation of study treatment, the PBO and RFX groups were broadly similar for several major clinical variables (**Table 1**). Nevertheless, some imbalances were observed. Total bilirubin levels were higher in the RFX group compared to placebo (median 82.0 vs. 56.0 μmol/L, *P* = 0.032). Similarly, liver transaminases (AST and ALT) were higher in the RFX arm than in the PBO arm (AST: 106 vs. 46 IU/L, *P* = 0.0052; ALT: 47.8 vs. 26.9 IU/L, *P* = 0.0071). GGT levels also tended to be higher in the RFX group (*P* = 0.052). By contrast, no clear between-group differences were observed for Child-Pugh score, renal function, CRP, or ascites severity at baseline. Despite these imbalances between the two arms, the longitudinal nature of our study allowed us to evaluate microbial changes by using each subject’s inclusion profile as their own control. Longitudinal Child-Pugh evolution and overall survival according to treatment arm are presented in **Supplementary Figures 1** and **2**, respectively.

**Figure 1:**
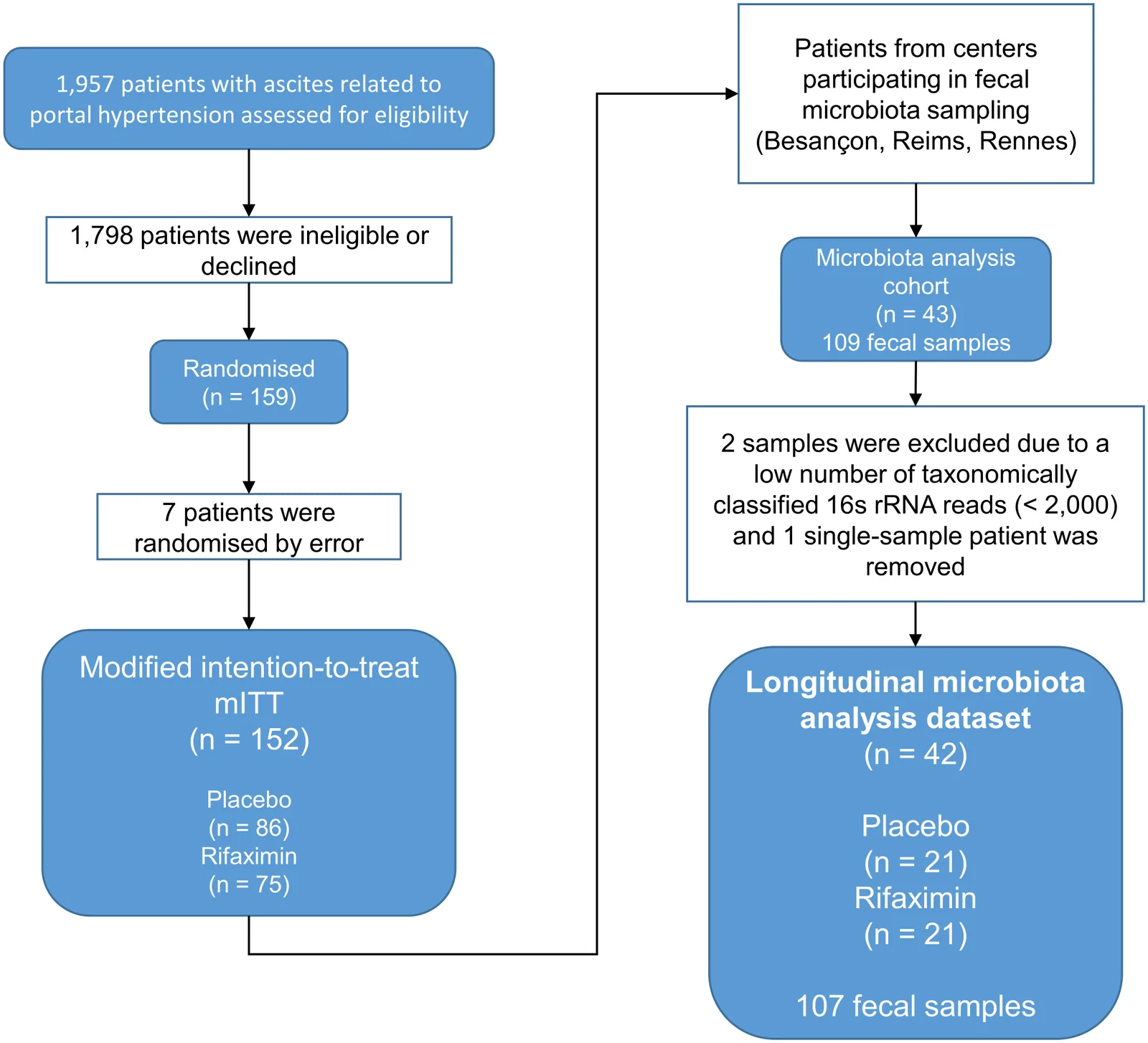
Flowchart of the study population. Of the 152 patients enrolled in the modified intention-to-treat (mITT) population of the original randomized trial, 43 patients from three recruiting centers had stool samples collected at one or more study visits. Overall, 109 fecal samples were processed for 16S rRNA gene sequencing. Following quality-control procedures, including removal of samples with <2,000 classified reads, 107 samples from 42 patients met inclusion criteria for downstream analyses.

**Table 1:** Baseline characteristics of patients in the placebo and rifaximin groups.

Characteristics	Total (n=42)	Placebo (n=21)	Rifaximin (n=21)	*P*
Age (years)^1^	59.3 (11.3)	60.1 (11.0)	58.4 (11.9)	0.6
Male gender^3^	34 (80.9)	16 (76.2)	18 (85.7)	0.7
Body mass index (kg/m^2^)^2^	26.6 (24.5-28.8)	26.5 (24.2-28.5)	26.6 (24.6-28.8)	0.8
Mean arterial pressure, mmHg^1^	82.4 (11.2)	80.4 (9.0)	84.3 (13.0)	0.3
Aetiology of liver disease^3^				1
Alcohol	41 (97.6)	21 (100.0)	20 (95.2)	
MASH	4 (9.5)	2 (9.5)	2 (9.5)	
Viral hepatitis B or C	2 (4.8)	1 (4.8)	1 (4.8)	
Severe alcoholic hepatitis^3^ suspected	13 (30.9)	2 (9.5)	11 (52.4)	0.002
Hepatocellular carcinoma^3^	2 (4.8)	1 (4.8)	1 (4.8)	1
Diabetes mellitus^3^	7 (16.7)	3 (14.3)	4 (19.0)	1
Child-Pugh score^2^	11.0 (10.0-12.0)	11.0 (10.0-11.0)	11.0 (10.0-12.0)	0.2
Total bilirubin (µmol/L)^2^	65.5 (49.0-104.0)	56.0 (48.0-68.0)	82.0 (62.0-116.0)	0.03
Prothrombin index (%)^2^	45.0 (41.0-57.0)	45.0 (42.0-54.0)	45.0 (38.0-58.0)	0.7
INR^1^	1.61 (0.24)	1.57 (0.22)	1.65 (0.25)	0.3
Serum albumin (g/L)^2^	27.0 (23.0-29.8)	28.4 (25.0-30.0)	27.0 (23.0-29.0)	0.3
Serum creatinine (µmol/L)^2^	66.3 (49.7-85.1)	63.1 (47.6-69.0)	71.9 (51.0-90.7)	0.1
Serum sodium (mmol/L)^1^	133.4 (4.7)	133.7 (5.3)	133.1 (4.2)	0.7
Platelet count (G/L)^2^	100.0 (84.0-141.0)	92.0 (80.0-150.0)	111.0 (87.5-140.0)	0.5
C-reactive protein (mg/L)^2^	15.1 (7.7-22.0)	9.7 (5.7-18.7)	17.0 (11.6-22.4)	0.06
Leucocytes/mm3^2^	6040 (5320-8200)	5800 (4570-7700)	6290 (5500-8200)	0.4
Total protein in ascites (g/L)^1^	10.7 (2.2)	10.1 (2.3)	11.2 (1.9)	0.1
Statins^3^	7 (16.7)	4 (19.0)	3 (14.3)	1
Proton-pump inhibitors^3^	26 (61.9)	13 (61.9)	13 (61.9)	1
Beta-blockers^3^	27 (64.3)	13 (61.9)	14 (66.7)	0.7
Lactulose^3^	24 (57.1)	14 (66.7)	10 (47.6)	0.2
Follow-up (days)^2^	365 (120-365)	365 (120-365)	365 (273-365)	0.8
AST (IU/L)^2^	77.0 (43.0-117.0)	46.0 (39.0-79.0)	106.0 (74.0-139.0)	0.005
ALT (IU/L)^1^	37.6 (25.3)	26.9 (13.9)	47.8 (29.6)	0.007
Gamma-GT (IU/L)^2^	97.0 (41.0-227.0)	57.0 (37.0-166.0)	146.0 (57.0-228.0)	0.05
Encephalopathy grade I/II^3^	3 (7.1)	2 (9.5)	1 (4.8)	1
Ascites^3^	42 (100.0)	21 (100.0)	21 (100.0)	1
Mild	5 (11.9)	2 (9.5)	3 (14.3)	
Large-volume	37 (88.1)	19 (90.5)	18 (85.7)	

MASH, metabolic dysfunction-associated steatohepatitis; MELD, model for end-stage liver disease.

Data are presented as ^1^mean (SD), ^2^median (IQR), or ^3^n (%), as appropriate.

Continuous variables were assessed with the Student’s t-test or Wilcoxon rank-sum test as appropriate. Categorical variables were assessed with the Chi-square test or Fisher’s test.

### 3.2 Rifaximin intake was associated with decreased gut microbiome α-richness

The decrease in available fecal samples over time in both treatment arms (**Figure 2A**) reflected predefined clinical discontinuation criteria, including death, liver transplantation, withdrawal of consent, investigator decision, loss to follow-up, and enrollment of ineligible subjects. Microbial richness and Shannon diversity were assessed using ASVs as species proxies across treatment groups and time points. At inclusion, median microbial richness was not different between groups, with 58.36 (IQR 46.65–73.30) ASVs in the PBO group and 65.65 (IQR 48.48–75.49) in the RFX group (Mann–Whitney test, *P* = 0.60; **Figure 2B**). In the PBO group, microbial richness remained stable over time (ART-ANOVA, *P* = 0.50). In contrast, microbial richness decreased over time in the RFX group (ART-ANOVA, *P* < 0.001), with reductions observed at M3, M6, and M12 compared to D1 (ART *post-hoc* tests, FDR = 0.01, 0.01, and 0.02, respectively). No changes in α-diversity were observed over time in either PBO or RFX groups (ART-ANOVA, both *P* = 0.20; **Figure 2C**). In a complementary treatment-by-time analysis, the overall interaction for Fisher richness approached statistical significance (P = 0.051). Planned interaction contrasts showed that the D1-to-M3 change differed significantly between the RFX and PBO arms (*P* = 0.006; FDR = 0.018), whereas the D1-to-M6 and D1-to-M12 contrasts were not significant after FDR correction (**Supplementary Table 3**). The treatment-by-time interaction was not significant for Shannon diversity (P = 0.183). Given the longitudinal decrease in microbial richness under RFX treatment (**Figure 2B**), we assessed whether RFX was associated with changes in overall community composition. Weighted UniFrac distances were computed and compared between inclusion and follow-up visits (M3, M6, M12) using a permANOVA test, excluding M18 to account for treatment discontinuation. In the PBO arm, community composition remained stable over time with no significant differences observed at any time point (**Table 2**). In contrast, a significant time effect was observed in the RFX arm at M3 (R²=0.059, *P* = 0.034 for Weighted; R²=0.060, *P* = 0.022 for Unweighted) and M6 (R²=0.096, *P* = 0.030 for Weighted; R²=0.096, *P* = 0.029 for Unweighted) compared to D1 (**Table 2**). However, this shift was transient, as community composition at M12 did not significantly differ from inclusion in the RFX group (*P* = 0.098 and *P* = 0.100 for Weighted and Unweighted UniFrac, respectively; **Table 2**). To determine taxa associated with the composition change induced by RFX treatment, a differential abundance analysis at the species level was performed (**Figure 3**). RFX treatment was associated with reduced relative abundances of 11 taxa, including *Veillonella* spp. (*V. atypica*, *V. dispar*, and *V. parvula)*, and *Escherichia coli*. Conversely, RFX was associated with increased relative abundances of eight taxa including *Dialister propionicifaciens*, *Fusicatenibacter saccharivorans*, and *Blautia massiliensis*. Relative abundance of these taxa did not change in the PBO arm. A complementary genus-level analysis identified seven significant contrasts involving six genera after FDR correction (**Supplementary Table 4**). Significant early contrasts were observed at M3 for *Methanobrevibacter*, *Morganella*, and *S5-A14a*, and at M6 for *Methanobrevibacter*. Additional contrasts were detected for *Clostridium sensu stricto 1* at M12 and for *Agathobacter* and *Akkermansia* at M18. M18 represents a post-treatment visit, approximately six months after RFX discontinuation.

**Figure 2:**
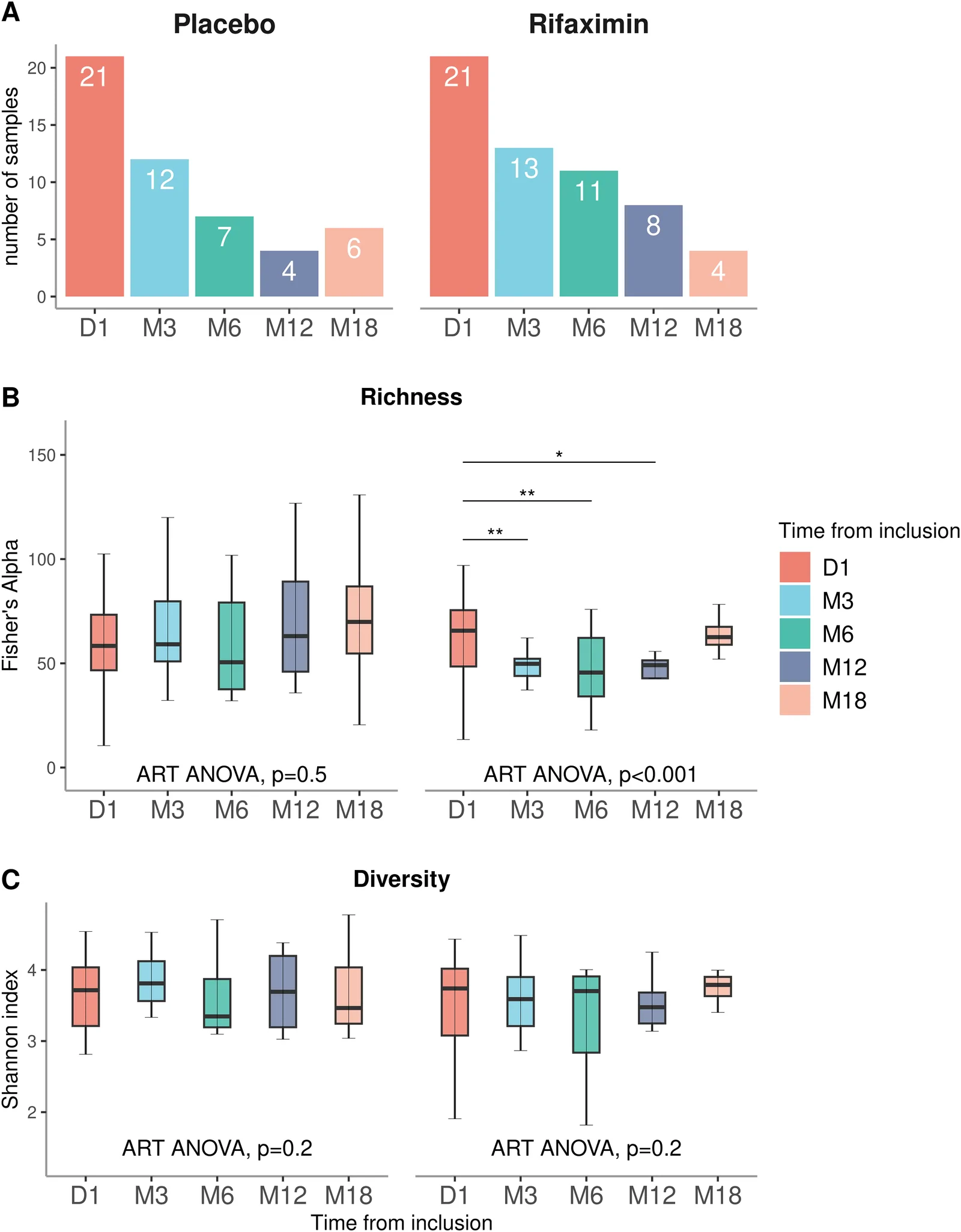
Longitudinal changes in gut microbiome α-diversity according to treatment arm. **(A)** Number of fecal samples available at each visit in the PBO and RFX arms. **(B)** Microbial richness and **(C)** Shannon diversity of the microbiome using ASVs as species proxies at inclusion (D1) and follow-up (M3, M6, and M12) visits. Statistical significance was assessed using ART-ANOVA with FDR-adjusted *post-hoc* tests (*FDR < 0.05; **FDR < 0.01).

**Table 2:** Comparison of β-diversity between follow-up visits (M3, M6, and M12) and inclusion (D1) in the PBO and RFX arms.

	Placebo		Rifaximin	
Time point comparison	Unweighted UniFrac	Weighted UniFrac	Unweighted UniFrac	Weighted UniFrac
M3 vs D1	R² = 0.032; *P* = 0.350	R² = 0.032; *P* = 0.355	R² = 0.060; *P* = 0.022	R² = 0.059; *P* = 0.034
M6 vs D1	R² = 0.042; *P* = 0.320	R² = 0.039; *P* = 0.336	R² = 0.096; *P* = 0.029	R² = 0.096; *P* = 0.030
M12 vs D1	R² = 0.066; *P* = 0.125	R² = 0.065; *P* = 0.125	R² = 0.057; *P* = 0.100	R² = 0.057; *P* = 0.098

Statistical significance was assessed using PERMANOVA tests (999 permutations) based on Unweighted and Weighted UniFrac distances. R² values represent the effect size (variance explained by time) and *P*-values indicate statistical significance.

**Figure 3:**
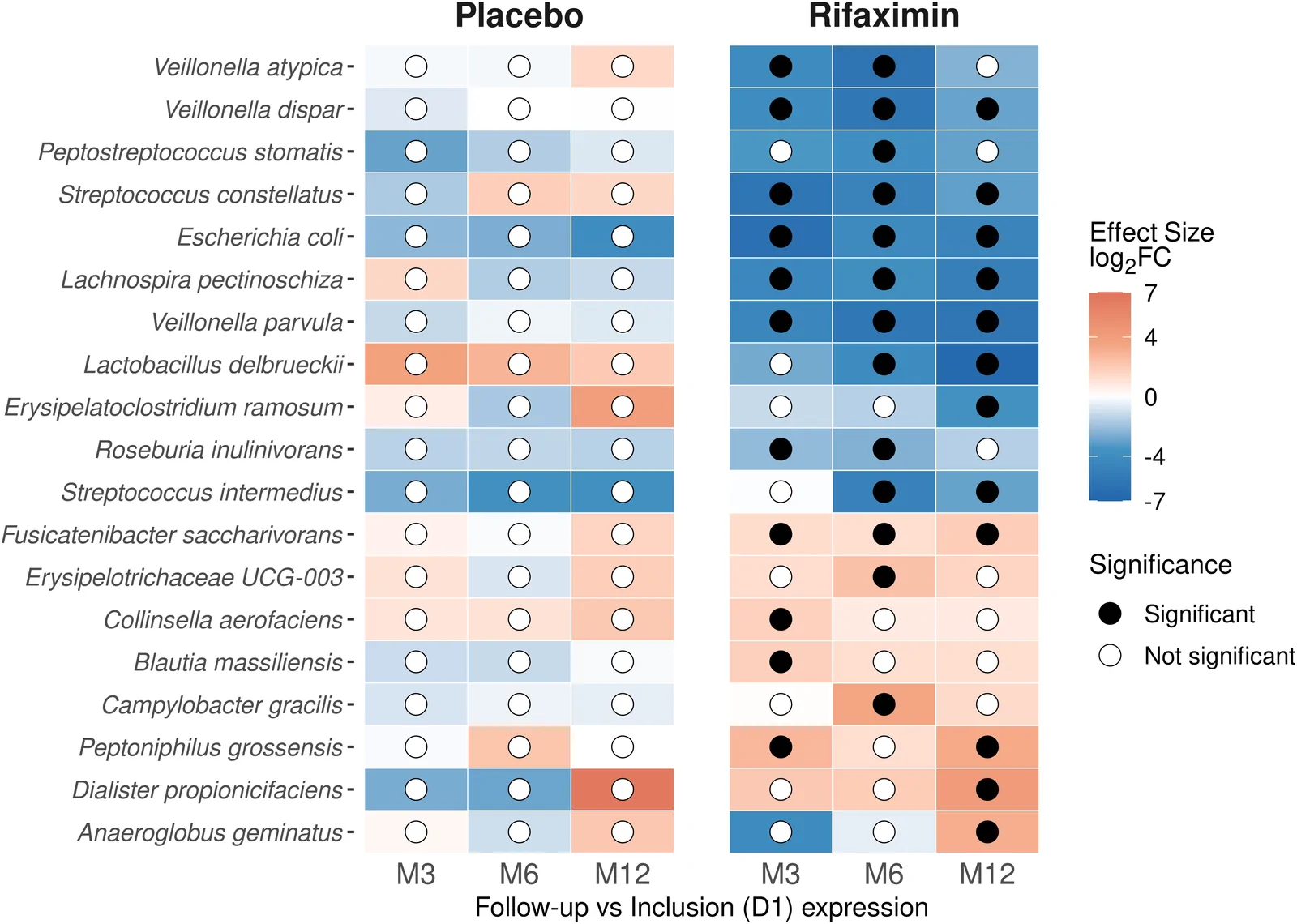
Rifaximin intake is associated with longitudinal changes in gut microbiome community composition and species-level abundances. Species-level differential abundance between inclusion (D1) and follow-up visits (M3, M6, M12) in the placebo and rifaximin arms, assessed using generalized linear mixed models. Colors indicate log_2_ fold changes relative to baseline, with blue and red representing decreased and increased abundances between follow-up and inclusion, respectively. Filled circles denote statistically significant associations after false discovery rate adjustment (*P* < 0.05).

### 3.3 Effect of rifaximin treatment on the emergence of antibiotic-resistant *E. coli*

Given that RFX exposure was associated with a decrease in the relative abundance of *E. coli* (**Figure 3**), and considering that *E. coli* resistance to RFX has been linked to prior RFX use (Kothary et al., 2013), we investigated whether RFX intake was associated with changes in the prevalence of phenotypically resistant *E. coli* strains. Using a culture-based approach, we determined the presence as well as the proportion of *E. coli* isolates resistant to rifampicin (RMP), a rifaximin analog, as well as to ciprofloxacin and cefotaxime, two major antibiotics. In the PBO group, the presence of patients harboring RMP-resistant (RMP-R) *E. coli* remained unchanged between inclusion and follow-up (Fisher’s exact test, *P* = 0.40; **Figure 4A**). In contrast, a higher number of patients in the RFX group harbored RMP-R *E. coli* at follow-up compared with inclusion (Fisher’s exact test, *P* < 0.001). Among fecal samples positive for RMP-R *E. coli*, the relative proportion of resistant isolates increased at follow-up in the RFX group (ART test, *P* = 0.02), whereas no change was observed in the PBO group (ART test, *P* = 1.00; **Figure 4B**). Finally, susceptibility testing of 105 RMP-R *E. coli* isolates revealed no cross-resistance to ciprofloxacin or cefotaxime (data not shown).

**Figure 4:**
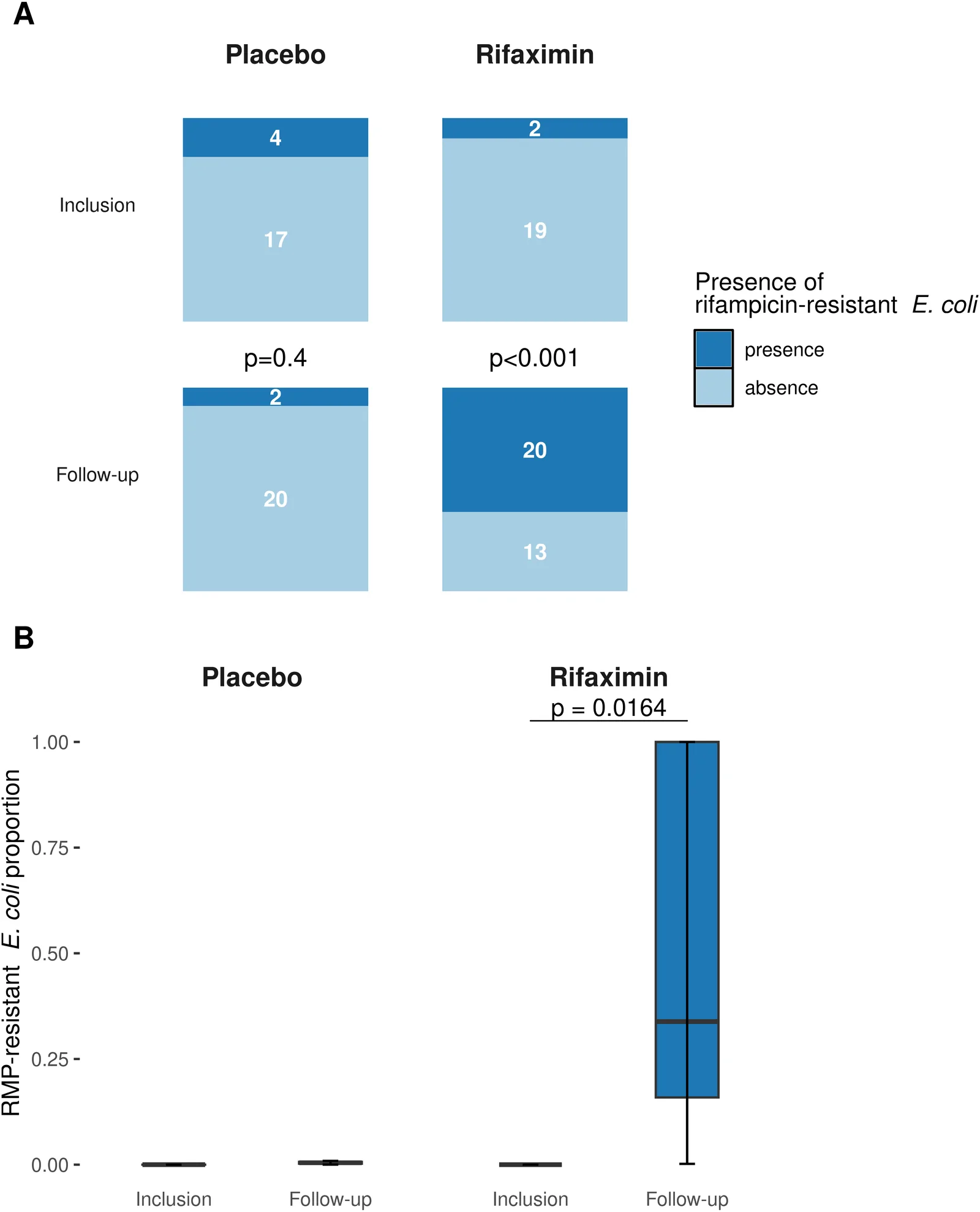
Rifaximin intake is associated with the emergence of rifampicin-resistant *Escherichia coli* in the gut. **(A)** Prevalence of rifampicin-resistant *E. coli* in stool samples at inclusion and follow-up. Numbers inside bars indicate the count of samples with resistant *E. coli* (dark blue) and without resistant *E. coli* (light blue) in each treatment arm. **(B)** Proportion of *E. coli* being resistant to rifampicin among fecal samples. Statistical significance was assessed using Fisher’s exact test **(A)** and ART models **(B)** for within group changes over time.

### 3.4 Exploratory analyses of gut microbiome composition and clinical outcomes

In the modified intention-to-treat analysis of the main trial (n = 152), RFX treatment was not associated with differences in 12-month overall survival or the incidence of cirrhosis-related complications (Thévenot et al., 2025). Comparable results were observed in the fecal donor sub-cohort (n = 42, **Supplementary Figures 2**, **3**, **Supplementary Tables 1**, **2**). Moreover, we found that Child-Pugh scores decreased during the follow-up visits compared to inclusion in both PBO and RFX arms (likelihood ratio test, *P* < 0.001) as presented in **Supplementary Figure 1**. In this context, we conducted exploratory analyses to investigate associations between bacterial species and clinical outcomes, independent of RFX exposure. First, the relationship between microbiome composition using weighted UniFrac distances and Child-Pugh scores was assessed. Child-Pugh score explained a modest but statistically significant proportion of the variance in community composition (permANOVA, R² = 0.064, *P* = 0.034), indicating an association between disease severity and commensal microbiome structure (**Figure 5A**). Then, species-level associations with disease severity were assessed using an ordinal mixed-effects regression model, with Child-Pugh score as the outcome, microbial abundance as the predictor, treatment arm as a covariate, and subject as a random effect. One model was fitted per species. Increases in Child-Pugh score, reflecting worsening disease severity, were associated with higher relative abundances of six taxa, including *Veillonella* spp. (*V. parvula*, *V. atypica*, and *V. dispar*) and *E. coli*. Conversely, decreases in Child-Pugh score over follow-up were associated with increased abundances of five commensal species (*Ruminococcus bromii*, *Blautia hansenii*, *Blautia obeum*, *Anaerostipes caccae*, and *Slackia isoflavoniconvertens*) (**Figure 5B**). These findings indicate that patients experiencing Child-Pugh improvement exhibited a progressive enrichment of commensal, health-associated taxa in their gut microbiome (Sasaki et al., 2022). Finally, the 11 species showing an association with changes in Child-Pugh score (**Figure 5**) were evaluated as potential predictors of outcome occurrence (SBP or overall survival) using their relative abundance at inclusion. Three of them showed significance in exploratory univariate competing risk analyses (**Figure 6**). Higher relative abundance of *E. coli* was associated with an increased 12-month cumulative incidence of SBP (Fine–Gray model, *P* = 0.028; **Figure 6A**). In contrast, lower 12-month cumulative SBP incidence was observed in patients with higher relative abundance of *B. obeum* (Fine–Gray model, *P* = 0.042; **Figure 6C**). Time-dependent receiver operating characteristic (ROC) analyses derived from the corresponding Fine–Gray models showed a moderate exploratory discriminatory potential of *E. coli* relative abundance for SBP occurrence at 6, 12, and 18 months (AUCs = 0.63–0.66; **Figure 6B**). *B. obeum* relative abundance demonstrated comparable discrimination for SBP risk across the same time horizons (AUCs = 0.62–0.77; **Figure 6D**). In survival analyses, higher relative abundance of *R. bromii* was associated with improved 12-month overall survival (univariate Cox proportional hazards model, *P* = 0.012; **Figure 6E**). Time-dependent ROC curves derived from the Cox model indicated moderate discrimination of *R. bromii* relative abundance for overall survival at 6, 12, and 18 months (AUCs = 0.61–0.79; **Figure 6F**). Results were consistent after additional adjustment for RFX treatment, with similar associations observed for both SBP incidence and overall survival (**Supplementary Table 5**).

**Figure 5:**
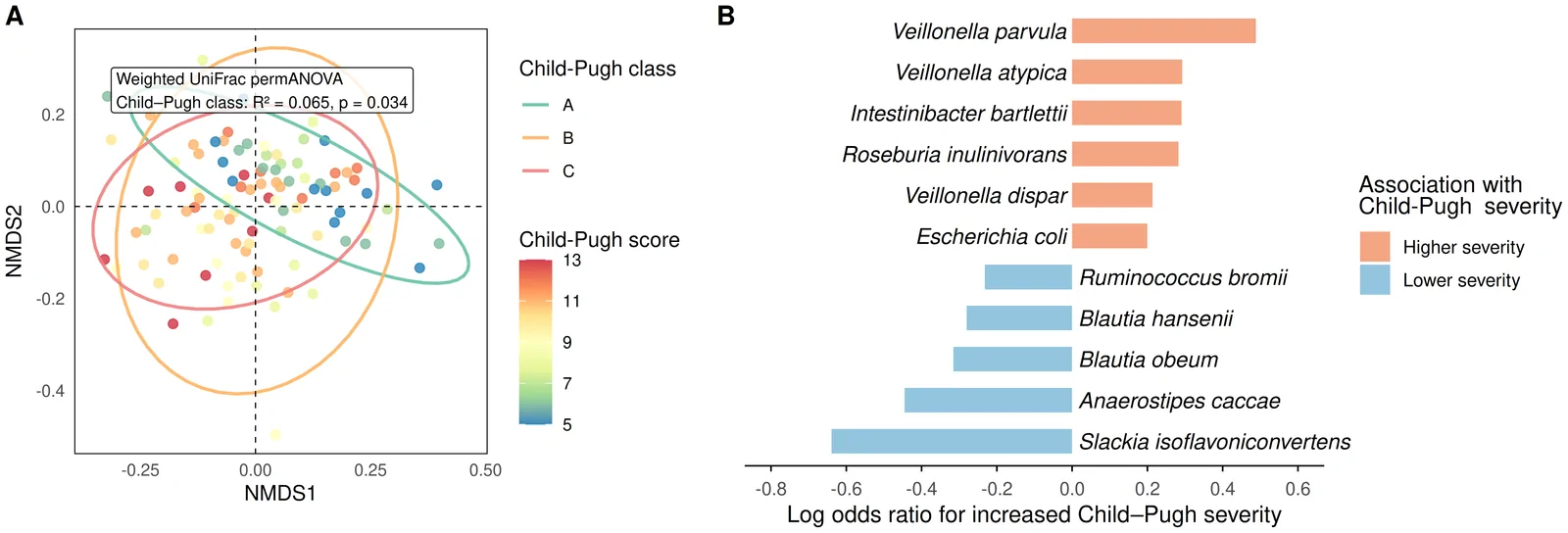
Association between gut microbiome composition and severity of liver disease. **(A)** Overall gut microbiota structure according to disease severity. Non-metric multidimensional scaling (NMDS) plot based on Weighted UniFrac distances. Each point represents an individual’s microbial profile, colored by Child-Pugh score (gradient from blue, lower severity, to red, higher severity). Ellipses represent the confidence areas for each Child-Pugh class **(A–C)**. PermANOVA results (R²=0.065, *P* = 0.034) indicate a statistically significant shift in global microbiome composition associated with increasing disease severity. **(B)** Species-level associations with disease severity identified using ordinal mixed-effects model. Bars represent log odds ratios associated with increasing Child-Pugh severity; positive values indicate taxa associated with greater disease severity whereas negative values indicate taxa associated with lower severity. Only species significantly associated with Child-Pugh score after false discovery rate (FDR) adjustment are shown.

**Figure 6:**
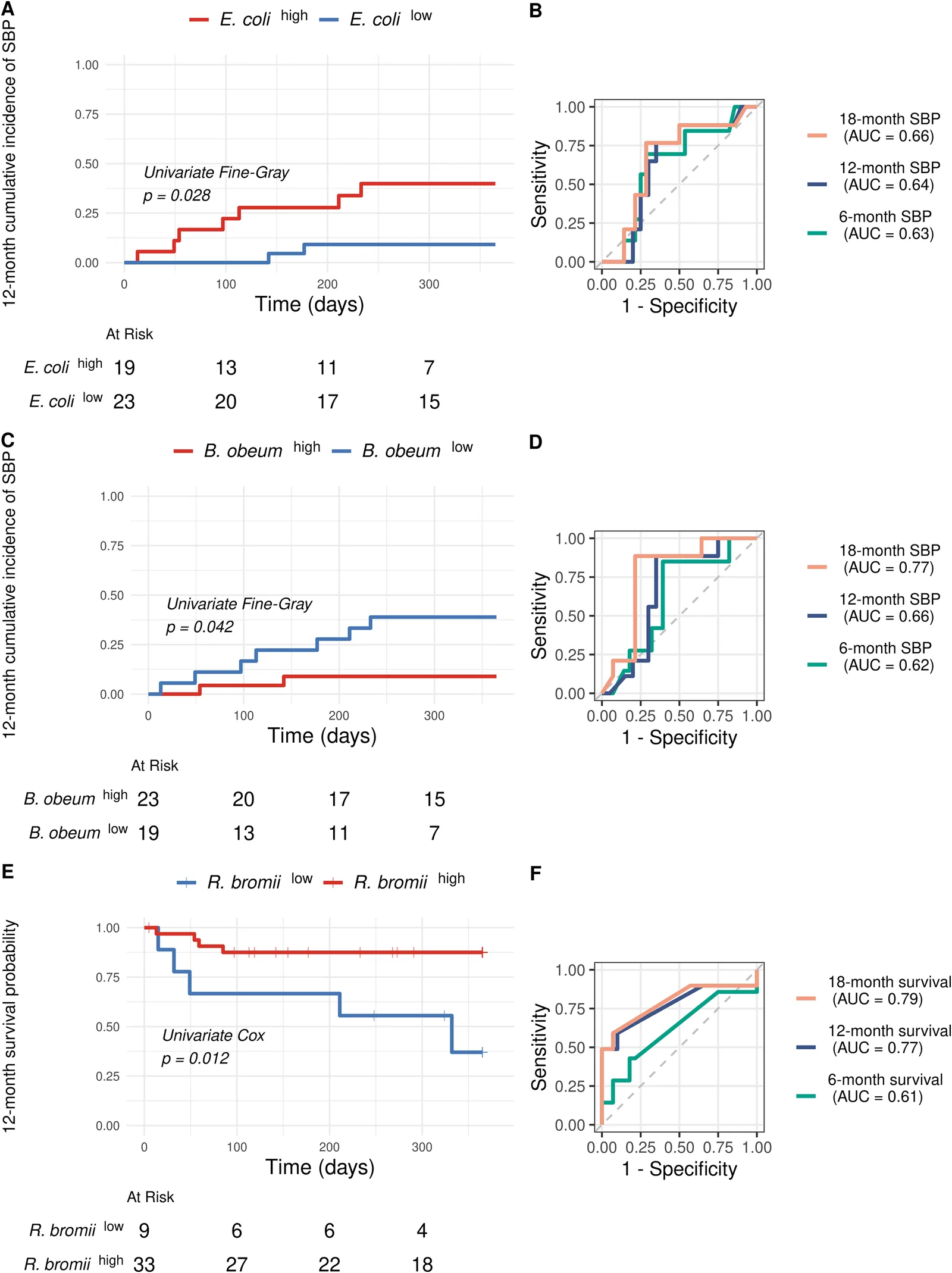
Stratification of spontaneous bacterial peritonitis (SBP) risk and overall survival according to cirrhosis severity–associated gut microbiome species. Cumulative incidence curves for SBP **(A, C)** and Kaplan–Meier survival curves for overall survival **(E)** were stratified according to the dichotomized relative abundances at inclusion (low *vs*. high) of selected species associated with Child-Pugh severity. SBP incidence was analyzed using univariate Fine–Gray competing risk models **(A, C)**, while overall survival was analyzed using univariate Cox proportional hazards models **(E)**. For each time-to-event analysis, time-dependent receiver operating characteristic (ROC) curves were generated to evaluate the discriminatory performance of species relative abundances at multiple time horizons. ROC analyses **(B, D)** corresponding to SBP incidence curves **(A, C)** are shown. ROC analysis **(F)** corresponding to overall survival **(E)** is shown. *P*-values correspond to the respective regression models. Censored observations are indicated by vertical tick marks on survival curves. Risk tables display the number of participants at risk and event-free at each time point in the low- and high-abundance groups.

## 4 Discussion

In this exploratory ancillary study of the ProPILA-Rifax trial, we investigated the longitudinal impact of RFX on the gut microbiome and the emergence of antibiotic resistance in patients with severe cirrhosis. We demonstrate that RFX reduces microbial richness, targeting cirrhosis-associated pathobionts, and promoting the emergence of RMP-resistant *E. coli*. Furthermore, independent of treatment, we identified microbial signatures associated with Child-Pugh scores, overall survival, and SBP incidence.

While some studies in other conditions report stable fecal richness under RFX, we observed a significant reduction in microbial richness in cirrhotic patients (**Figures 1**, **2B**). These results suggest a selective loss of rare luminal taxa as well as commensal communities under RFX pressure, confirmed using Unweighted and Weighted UniFrac analyses (**Table 2**). Differential analyses showed that RFX reduced the abundance of several pathobionts (e.g., E. coli, Streptococcus spp., and Veillonella spp.), suggesting that RFX can modulate microbiome ecological balance towards a more healthy state in the context of advanced cirrhosis. Even though RFX has traditionally been considered low risk for inducing resistance (Kuo et al., 2025), long-term and repeated use should be monitored as daptomycin-resistant enterococci have been reported in patients treated with RFX (Turner et al., 2024). Here, we confirmed that RFX favors the emergence of RMP-resistant E. coli (**Figure 4**), a pattern previously observed in patients suffering from inflammatory bowel disease (Kothary et al., 2013). As E. coli is the predominant cause of SBP in cirrhotic patients (Soriano et al., 1995), this finding raises concerns about potential resistance selection. Nevertheless, we found that RFX did not lead to co-resistance or multi-resistance to key antibiotics such as third-generation cephalosporins or fluoroquinolones, supporting its cautious use in targeted clinical scenarios. These microbiological findings perfectly align with recent large-scale clinical observations from the global CLEARED consortium, which concluded that prior RFX use does not increase the risk of drug-resistant organism infections after accounting for disease severity (Bajaj et al., 2026). Together, these data suggest that while RFX drives specific localized resistance, it does not broadly compromise first-line antibiotic therapies.

Primary analyses of the ProPILA-Rifax were replicated on this sub-cohort, and significant differences in complication incidence and survival probability among patients in the RFX group compared with controls (**Supplementary Figures 2**, **3**) were also not observed. A significant decrease in Child-Pugh score in both arms during follow-up compared to inclusion was observed (**Supplementary Figure 1**). This phenomenon may reflect the combined effects of optimized management of cirrhosis-related complications within the trial setting, along with a potential reduction in alcohol consumption. We therefore investigated bacterial species associated with clinical outcomes (*i.e.*, disease severity, overall survival, and SBP) independently of RFX exposure. Mixed-effects models showed that lower Child-Pugh scores were associated with reduced abundances of *E. coli* and *Veillonella* spp. (**Figure 5B**). Previous studies have reported enrichment of these taxa in patients with advanced cirrhosis (Rocco et al., 2021; Won et al., 2022). Progressive oralization of the gut microbiota can occur in decompensated cirrhosis and acute-on-chronic liver failure. This phenomenon, characterized by an overlap between oral and gut microbiomes due to the ectopic expansion of oral-origin bacteria within the intestinal ecosystem, has been elucidated in a recent metagenomic sequencing study of paired saliva and fecal samples (Horvath et al., 2019). Taxa such as *Veillonella* spp. and *Streptococcus* spp. were identified as important features of the oralization observed in this cohort (Horvath et al., 2019; Bajaj et al., 2021; Lee et al., 2025). Our results demonstrate that Child-Pugh score reduction positively correlates with the attenuation of gut flora oralization (**Figure 5**). Liver improvement may reflect multiple factors, including optimized management of cirrhosis-related complications and potential changes in alcohol consumption during follow-up. This ecological transition—characterized by a decline in oral pathobionts (e.g., *Veillonella* spp.) and a resurgence of short-chain fatty acid (SCFA)-producing keystone species such as *Ruminococcus bromii*, *Blautia obeum*, *Anaerostipes caccae*, and *Slackia isoflavoniconvertens* (Bloom et al., 2021; Cerqueira et al., 2022; Sakai et al., 2024) likely reflects a multifactorial process. Beyond associations, we hypothesize a mechanistically driven feedback loop where SCFAs (acetate, propionate, and butyrate) act as metabolic mediators (Qin et al., 2025). Earlier studies have shown that SCFAs can signal through the GPR43 receptor, inducing potassium efflux and membrane hyperpolarization. This cascade promotes activation of the NLRP3 inflammasome, a key sensor involved in stress responses, which contributes to preserving intestinal barrier integrity by supporting epithelial repair and enhancing cell survival under damaging conditions (Li et al., 2024). This pattern is consistent with prior work linking a history of overt HE to depletion of multiple SCFA-associated species and lower fecal SCFA concentrations, supporting the view that restoration of anaerobic, SCFA-producing networks may accompany improved clinical states (Wang et al., 2023). In this study, several commensal taxa associated with lower Child-Pugh scores, including *Ruminococcus bromii* and *Blautia obeum*, have previously been linked to SCFA-producing microbial networks. However, SCFAs were not measured in the present study, and their concentrations cannot be reliably inferred from 16S rRNA sequencing data. Therefore, a potential contribution of SCFA metabolism remains hypothetical and will require validation using targeted metabolomic approaches.

We demonstrate that inclusion abundance of *R. bromii* discriminates survivors from non-survivors (**Figure 6E**). To our knowledge, this represents the first evidence in severe cirrhosis linking a single keystone species to improved survival. Similarly, higher *B. obeum* levels were associated with a lower incidence of SBP (**Figure 6C**), aligning with murine models where *Blautia* spp. maintain colonic mucus integrity under environmental stress (Liu et al., 2021). Given that *E. coli* is the primary driver of SBP (Soriano et al., 1995), the parallel depletion of mucus-shielding commensals and the expansion of *Enterobacterales* establishes a logical ecological mechanism for ascitic infections. The association between *R. bromii* and survival is consistent with experimental studies suggesting potential metabolite-mediated effects, including acetate-related pathways; however, this mechanism was not directly assessed in our study. Ultimately, the restoration of *Blautia*-driven mucus integrity—preventing translocation—combined with *R. bromii*-mediated anti-fibrotic signaling, may represent a dual ecological signature of improved clinical outcomes (Li et al., 2025).

We acknowledge several limitations. First, the microbiome subcohort was small (n = 42), with progressive attrition at later follow-up visits and baseline imbalances in bilirubin and transaminase levels. Although randomization supports comparisons between RFX and placebo, residual imbalance remains possible in this small ancillary subset. Baseline information was available for several microbiota-modifying factors, including proton-pump inhibitor use, lactulose use, and diabetes; however, comprehensive longitudinal information on medication exposure, diet, and alcohol consumption was not consistently available, precluding reliable adjustment for these potential confounders. This limitation is particularly relevant to the exploratory associations between microbiota features and clinical outcomes. Finally, fecal SCFA concentrations were not measured, precluding direct assessment of the proposed metabolite-mediated mechanisms. These exploratory findings therefore require validation in larger prospective cohorts. Despite these constraints, our findings address a critical gap as the management of decompensated cirrhosis shifts from broad-spectrum bacterial suppression toward targeted ecological restoration. While fecal microbiota transplantation (FMT) remains a promising avenue in managing HE, recent THEMATIC trial suggests that FMT therapeutic efficacy depends on the recipient’s inclusion abundance of *Lachnospiraceae* and *Ruminococcaceae* (Bajaj et al., 2025). In this context, our study provides novel evidence that the inclusion abundance of a single keystone species, *Ruminococcus bromii*, serves as a prognostic determinant for survival in severe cirrhosis. By identifying specific, actionable taxa associated with Child-Pugh improvement and survival, our findings establish a framework for future preclinical and clinical studies aimed at engineering precision, function-restoring microbiome interventions in advanced liver disease.

## Data Availability

The datasets presented in this study can be found in online repositories. The names of the repository/repositories and accession number(s) can be found below: https://www.ebi.ac.uk/ena, ERP167667.
