# Supplementary material for: Gut microbial dynamics and antibiotic resistance during rifaximin therapy in severe cirrhosis: a ProPILA-Rifax ancillary study
Source: Front Cell Infect Microbiol. 2026 Sep 29;16:1930709. doi: 10.3389/fcimb.2026.1930709 (PMC13658500; doi:10.3389/fcimb.2026.1930709)
Supplement: Supplementary file 1 [file Table1.docx]

Supplementary Material

## 1. Supplementary Figures

**Supplementary Figure 1.** Longitudinal evolution of liver disease severity. Child–Pugh score overtime (D1, M3, M6, and M12) in the microbiome subcohort (n=42). Individual values are shown, colored according to Child–Pugh class (A, B, C). Boxplots represent median and interquartile range. Overall temporal differences of Child-Pugh score between inclusion (D1) and follow-up visits (M3, M6, and M12) were assessed using a likelihood-ratio test (LRT) derived from a mixed-effects model with patients as random effects (∗p<0.05, ∗∗p<0.01, ∗∗∗p<0.001).

**Supplementary Figure 2.** Overall survival according to treatment arm. Kaplan–Meier estimates of overall survival in patients receiving placebo or rifaximin during follow-up. Tick marks indicate censored observations. Survival distributions were compared using the log-rank test (p = 0.96). Risk tables display the number of participants at risk and event-free at each time point in the Placebo and Rifaximin groups. Corresponding survival estimates at months 3, 6, and 12 are provided in **Supplementary Table 1**.

**Supplementary Figure 3**. Cumulative incidence of spontaneous bacterial peritonitis. Cumulative incidence curves of spontaneous bacterial peritonitis (SBP) according to treatment arm (placebo vs rifaximin). The analysis was performed using a competing risks framework. Differences between groups were assessed using Gray’s test (p = 0.6). Risk tables display the number of participants at risk and event-free at each time point in the Placebo and Rifaximin groups. Corresponding cumulative incidence estimates at months 3, 6, and 12 are reported in **Supplementary Table 2**.

## 2. Supplementary Tables

**Supplementary Table 1**. Overall survival estimates. Estimated overall survival rates (%) with 95% confidence intervals at months 3, 6, and 12 according to treatment arm in the microbiome subcohort (n = 42). Survival curves were compared using the log-rank test. This table complements the Kaplan–Meier curves shown in **Supplementary Figure 2**.

| **Arm** |  | **Follow-up visit** |  | ***P*-value**^1^ |
| --- | --- | --- | --- | --- |
|  | **Month 3** | **Month 6** | **Month 12** |  |
| Placebo | 81 (66, 100) | 76 (60, 96) | 75 (59, 97) | >0.9 |
| Rifaximin | 90 (77.8, 100) | 85.0 (70, 100) | 72.9 (55, 96) |  |
| ^1^Log-rank test | | | | |

**Supplementary Table 2**. Cumulative incidence of spontaneous bacterial peritonitis. Cumulative incidence of spontaneous bacterial peritonitis (%) with 95% confidence intervals at months 3, 6, and 12 according to treatment arm. Group comparisons were performed using Gray’s test. This table complements the cumulative incidence curves presented in **Supplementary Figure 3**.

| **Arm** |  | **Follow-up visit** |  | ***P*-value**^1^ |
| --- | --- | --- | --- | --- |
|  | **Month 3** | **Month 6** | **Month 12** |  |
| Placebo | 4.8 (0.30, 20) | 24 (8.5, 45) | 30 (12, 50) | 0.6 |
| Rifaximin | 14 (3.4, 33) | 19 (5.7, 38) | 39 (18, 60) |  |
| ^1^Gray's test | | | | |

**Supplementary Table 3**. Treatment-by-time interaction contrasts for microbial richness. Planned treatment-by-time interaction contrasts from the aligned rank transform mixed-effects analysis of Fisher's alpha richness. The model included treatment arm, time, their interaction, and subject as a random effect. P-values were FDR-adjusted across the three planned D1-to-follow-up contrasts. The overall treatment-by-time interaction approached statistical significance (*P* = 0.051). The D1-to-M3 interaction contrast remained significant after FDR correction. M18 was excluded because rifaximin treatment was discontinued at M12. FDR, false discovery rate; PBO, placebo; RFX, rifaximin.

| **Arm comparison** | **Time contrast** | **ART contrast Estimate** | **SE** | ***P*-value**^1^ | **FDR** |
| --- | --- | --- | --- | --- | --- |
| Placebo - Rifaximin | D1→M3 | -27.27 | 9.57 | 0.00613 | 0.0184 |
| Placebo - Rifaximin | D1→M6 | -6.81 | 11.00 | 0.53869 | 0.5387 |
| Placebo - Rifaximin | D1→M12 | -11.03 | 13.30 | 0.41054 | 0.5387 |
| ^1^Aligned rank transform mixed-effects model | | | | | |

**Supplementary Table 4.** Significant genus-level treatment-by-time interaction contrasts. Genus-level abundances were assessed using zero-inflated negative-binomial generalized linear mixed models including treatment arm, time, their interaction, sequencing depth as an offset, and subject as a random effect. Planned interaction coefficients compared the D1-to-follow-up changes between the RFX and placebo arms. Only significant FDR-adjusted contrasts are displayed. The time contrast represents the difference in longitudinal change between the RFX and placebo arms from D1 to the indicated follow-up visit. Positive log2 interaction FC values indicate a greater increase, or smaller decrease, under RFX relative to placebo; negative values indicate a greater decrease, or smaller increase, under RFX relative to placebo.

| **Genus** | **Time contrast** | **log2 FC** | ***P*-value¹** | **FDR²** |
| --- | --- | --- | --- | --- |
| *Agathobacter* | D1→M18 | 13.97 | <0.001 | < 0.001 |
| *Akkermansia* | D1→M18 | -11.07 | <0.001 | 0.01273 |
| *Clostridium sensu stricto 1* | D1→M12 | 6.77 | <0.001 | 0.00269 |
| *Methanobrevibacter* | D1→M3 | -12.22 | <0.001 | 0.03288 |
| *Methanobrevibacter* | D1→M6 | -9.78 | <0.001 | 0.03652 |
| *Morganella* | D1→M3 | -11.05 | <0.001 | < 0.001 |
| *S5-A14a* | D1→M3 | -7.04 | <0.001 | 0.03288 |

^1^Wald z-test

**Supplementary Table 5**. Associations between bacterial species abundance and clinical outcomes (provided as a separate Excel file). This table summarizes the results of survival analyses assessing whether high versus low abundances at inclusion of specific bacterial taxa are associated with (i) the 12-month cumulative incidence of spontaneous bacterial peritonitis (SBP) using Fine–Gray competing-risk models, and (ii) 12-month overall survival using Cox proportional hazards models. For each species, both univariate models and multivariable models adjusted for treatment allocation were fitted. Species-specific abundance thresholds (“Cutoff”) were determined using a grid-search AIC-minimizing procedure. Patients were stratified into low- and high-abundance groups based on this cutoff, and sample sizes and event counts are reported for each group. Effect sizes are expressed as sub-distribution hazard ratios (sHR) for Fine–Gray models or hazard ratios (HR) for Cox models, with corresponding 95% confidence intervals and P-values. Models explicitly state whether they are adjusted or unadjusted for rifaximin effects.

**Column Definitions**

**Genus**	Bacterial genus associated with the clinical endpoint.

**Species**	Bacterial species associated with the clinical endpoint.

**Outcome**	Clinical outcome evaluated: either 12-month cumulative incidence of SBP (competing-risk endpoint) or 12-month overall survival.

**Model**	Statistical model used: univariate Fine–Gray, Fine–Gray adjusted for RFX treatment, univariate Cox PH, or Cox PH adjusted for RFX treatment.

**Cutoff**	Abundance threshold separating “low” and “high” groups. Determined using grid-search minimizing AIC.

**Effect_type**	Indicates whether the reported effect is a sub-distribution hazard ratio (sHR, Fine–Gray) or hazard ratio (HR, Cox PH).

**Effect**	Estimated effect size (sHR or HR). Values above 1 indicate increased risk; values below 1 indicate reduced risk.

**CI_low**	Lower bound of the 95% confidence interval for the effect estimate.

**CI_high**	Upper bound of the 95% confidence interval for the effect estimate.

**Pvalue**	P-value assessing the statistical significance of the association.

**N_low**	Number of patients with bacterial abundance below the cutoff.

**N_high**	Number of patients with bacterial abundance above the cutoff.

**Events_low**	Number of clinical events (SBP or deaths) occurring in the low-abundance group.

**Events_high**	Number of clinical events occurring in the high-abundance group.
